# Alteration of the fecal microbiota in Chinese patients with *Schistosoma japonicum* infection

**DOI:** 10.1051/parasite/2020074

**Published:** 2021-01-08

**Authors:** Yanyan Jiang, Zhongying Yuan, Yujuan Shen, Bruce A. Rosa, John Martin, Shengkui Cao, Yanjiao Zhou, Makedonka Mitreva, Jianping Cao

**Affiliations:** 1 National Institute of Parasitic Diseases, Chinese Center for Disease Control and Prevention 200025 Shanghai PR China; 2 Chinese Center for Tropical Diseases Research 200025 Shanghai PR China; 3 World Health Organization Collaborating Centre for Tropical Diseases 200025 Shanghai PR China; 4 Key Laboratory of Parasite and Vector Biology, National Health Commission of the People’s Republic of China 200025 Shanghai PR China; 5 McDonnell Genome Institute, Washington University in St Louis St Louis 63001 MO USA; 6 Department of Medicine, UConn Health Farmington 06023 CT USA; 7 Division of Infectious Diseases, Department of Medicine, Washington University School of Medicine St Louis 63001 MO USA

**Keywords:** *Schistosoma japonicum*, 16s rDNA, Infectious disease, Fecal microbiome, Enterotype

## Abstract

*Schistosoma japonicum* infection causes pathological injury to the host. Multiple studies have shown that intestinal helminth infection causes dysbiosis for the gut microbial community and impacts host immunology. However, the effect of acute *S. japonicum* infection on the gut microbiome structure (abundance and diversity) is still unclear. We collected fecal samples from healthy and infected patients from a single hospital in Hunan Province, China. The bacterial community was analyzed using 16S ribosomal RNA gene sequencing of the V4 hypervariable region using the HiSeq platform. Compared with healthy subjects, infected patients exhibited an increase in relative abundance of the TM7 phylum. At the genus level, there were seven differentially abundant genera between groups. The most significant finding was a *Bacteroides* enterotype in patients with acute schistosomiasis. These results suggest that *S. japonicum* infection has a significant effect on microbiome composition characterized by a higher abundance of the TM7 phylum and development of a *Bacteroides* enterotype.

## Abstract (fr)

L’infection à *Schistosoma japonicum* provoque des lésions pathologiques chez l’hôte. Plusieurs études ont montré qu'une infection intestinale par les helminthes provoque une dysbiose de la communauté microbienne intestinale et a un impact sur l’immunologie de l’hôte. Cependant, l’effet de l’infection aiguë à *S. japonicum* sur la structure du microbiome intestinal (abondance et diversité) n’est toujours pas clair. Nous avons collecté des échantillons fécaux de patients sains et infectés dans un hôpital de la province du Hunan, en Chine. La communauté bactérienne a été analysée par séquençage du gène de l’ARN ribosomal 16S de la région hypervariable V4 en utilisant la plateforme HiSeq. Par rapport aux sujets sains, les patients infectés ont présenté une augmentation de l’abondance relative du phylum TM7. Au niveau du genre, il y avait sept genres différentiellement abondants entre les groupes. La découverte la plus significative était un entérotype *Bacteroides* chez les patients atteints de schistosomiase aiguë. Ces résultats suggèrent que l’infection à *S. japonicum* a un effet significatif sur la composition du microbiome caractérisé par une plus grande abondance du phylum TM7 et le développement d’un entérotype *Bacteroides*.

## Introduction

Schistosomiasis remains a devastating and highly prevalent neglected tropical disease that is endemic mainly in poor and undeveloped regions [50]. Of the three major pathogenic species causing schistosomiasis, *Schistosoma japonicum* is responsible for human and animal infections in parts of East and Southeast Asia, primarily China, the Philippines, and Indonesia [58]. Adult *S. japonicum* worms inhabit the mesenteric veins of the gut and trigger a cellular immune response in the host when eggs are released, which leads to a wide range of clinical manifestations including gut inflammation [25]. This also affects the gut microbiota [28].

The human gut is colonized by an enormous community of microbes, termed the microbiota [8], which impacts the host immune system. The gut microbiota interacts with interleukin-17 to induce T helper cell differentiation in the lamina propria of the small intestine to drive autoimmune disease [53]; the microbiota also provides resistance to colonization by enteric pathogens [4]. Alterations in the human microbiome have been associated with a range of conditions in the developed world, including diabetes [55], non-alcoholic fatty liver disease (NAFLD) [45], inflammatory bowel disease (IBD), cardiovascular disease, cancer [3], and refractory *Clostridium difficile* infection [38]. Microbiome changes have been observed in people with parasitic nematode infections, including higher relative abundance of Paraprevotellaceae in patients infected with *Trichuris trichiura* [34] and an increased abundance of Sphingobacteria, Deltaproteobacteria*,* and Erysipelotrichia after deworming [44]. Moreover, these microbiome changes have been observed in children with parasitic protozoa infections [23, 40, 52]. Children [32] and adults [1] infected with *S. haematobium* had altered gut microbiota, urogenital schistosomiasis, and altered bladder pathologies. In addition, a mouse model of *S. mansoni* infection showed that depletion of gut bacteria resulted in a reduction in schistosome egg excretion, an alteration in the specific immune response [28], and an alteration in inflammatory response and gut pathology caused by specific bacteria [30].

Studies analyzing the effect of *S. japonicum* infection on the gut microbiota are lacking. We directly addressed this issue by examining the intestinal microbial community during acute *S. japonicum* infection, and identifying associations between bacteria and enterotypes with acute schistosomiasis in an epidemic area of China.

## Materials and methods

### Ethics statement

All experiments were approved by the Ethics Committee of the National Institute of Parasitic Diseases, Chinese Center for Disease Control and Prevention (No. 2015-011). All participants were informed of the objectives, procedures and potential risks of the study. Written informed consent forms were personally signed by all adult subjects. The personal information of all the participants has been kept confidential.

### Subjects and sample collection

Twenty-six participants living in the same geographical area were included in this cross-sectional study (Table 1). They were local male fishermen without hepatitis B or C infections, who had not received any medical therapy within the previous three months. All of the study participants were >30 years old, and had no other parasitic infection. The study was conducted between September and December 2016.

**Table 1:** Participant profiles.

No. participant	Age (year)	Group	Kato-Katz method	IHA		
				1:2	1:10	1:20
SJ_1	57	≥50-year-old	+	–	+	+
SJ_2	48	<50-year-old	+	–	+	–
SJ_3	52	≥50-year-old	+	–	+	–
SJ_4	39	<50-year-old	+	–	+	–
SJ_5	46	<50-year-old	+	–	+	+
SJ_6	44	<50-year-old	+	–	+	+
SJ_7	49	<50-year-old	+	+	–	–
SJ_8	48	<50-year-old	+	+	–	–
SJ_9	52	≥50-year-old	+	–	+	+
SJ_10	66	≥50-year-old	+	+	–	–
SJ_11	48	<50-year-old	+	+	–	–
C_1	58	≥50-year-old	–	–	–	–
C_2	46	<50-year-old	–	–	–	–
C_3	50	≥50-year-old	–	–	–	–
C_4	64	≥50-year-old	–	–	–	–
C_5	51	≥50-year-old	–	–	–	–
C_6	42	<50-year-old	–	–	–	–
C_7	59	≥50-year-old	–	–	–	–
C_8	45	<50-year-old	–	–	–	–
C_9	56	≥50-year-old	–	–	–	–
C_10	59	≥50-year-old	–	–	–	–
C_11	68	≥50-year-old	–	–	–	–
C_12	56	≥50-year-old	–	–	–	–
C_13	60	≥50-year-old	–	–	–	–
C_14	67	≥50-year-old	–	–	–	–
C_15	42	<50-year-old	–	–	–	–

Eleven patients were initially screened for *Schistosoma* spp. eggs in feces using the Kato-Katz method [31] and by quantifying levels of indirect hemagglutination antibody (IHA) in the serum. They also had a fever or fatigue accompanied by tenderness in the liver region, and had been in contact with cercariae in water in the previous three months. They had no other helminth eggs (e.g., hookworm, roundworm, whipworm, pinworm, or *Taenia*) according to microscopic examination, but fecal samples were found to contain *Schistosoma* spp. eggs. Moreover, molecular examinations were not indicative of any emerging and important protozoa (such as *Blastocystis* spp., *Giardia intestinalis*, *Entamoeba* spp., *Cryptosporidium* spp., *Enterocytozoon bieneusi*, and *Cyclospora cayetanensis*). After sample collection, all infected patients were treated with praziquantel.

The 15 healthy subjects (control) did not display clinical symptoms of schistosome infection and yielded negative laboratory results according to the Kato-Katz method and IHA test, and they had no history of schistosomiasis.

A total of 26 fresh fecal samples (15 controls, 11 patients) were stored in 2.0-mL Eppendorf tubes and frozen at −80 °C until DNA extraction.

### DNA extraction

DNA was extracted from a frozen aliquot (200 mg) of each fecal sample using a QIAamp DNA Mini Stool Kit (Qiagen, Valencia, CA, USA). DNA concentration was measured by a Qubit 2 (Invitrogen) and its molecular size was estimated by agarose gel electrophoresis. DNA libraries were constructed according to the manufacturer’s instructions (Illumina). The V4 region of the 16S rDNA gene was amplified using the 515F forward (5′ – GTGCCAGCMGCCGCGGTAA – 3′) and 806R reverse primers (5′ – GGACTACHVGGGTWTCTAAT – 3′) (BGI company, China). Sequencing was performed using Illumina HiSeq (Illumina, paired end, 250 bp reads).

### Analytical processing and annotation of the 16S rRNA sequences

Sequence reads were joined using fastq-join and quality filtered (phred score *Q* ≥ 25). Sequences were assembled using the FLASh assembler [36]. Assembled reads were filtered to remove sequences shorter than 200 bp and any sequences containing fewer than 42 N-free 8-mers. Chimera sequences were identified and removed using ChimeraSlayer, with default parameters [16]. As a result, the total dataset comprised approximately 0.9 million reads, and the number of reads of individual samples ranged from 30,387 to 40,304. Taxonomic calls were assigned using the classify.seqs program with the Mothur 16S rRNA gene data processing pipeline [46]. The Ribosomal Database Project Naïve Bayesian Classifier (version 2.5 with training set 9 [9]) (Release9 201203[10]) was used with a 0.5 confidence level. Reads with <0.5 confidence of classification was considered to be “unclassified” at a given taxonomical level.

### Statistical analysis

Microbiota richness was determined using the Chao1 index and Shannon’s diversity index. These indices were calculated with QIIME and displayed with R software (version 2.15.3), and the Kruskal–Wallis test was used for comparison between groups. Beta diversity analysis (i.e., differences in samples in terms of bacterial community composition) was visualized using Principal Coordinates Analysis (PCoA) with the “cmdscale” function in R. Samples were clustered according to relative taxa abundance values across all taxa (i.e., read counts normalized by total reads mapped per sample) with the “hclust” function in R to interpret the distance matrix using complete linkage. DESeq2 was used to identify specific taxa that are significantly different between patients and healthy subjects [35] (unpaired differential analysis) in R for abundance testing of differential taxa. DESeq2 was the preferred approach based on its negative binomial statistical design and high performance over a full range of effect sizes, replicate numbers, and library sizes [39]. All DESeq2 input data (read counts) and output results are available in Supplemental Table 1. The phylum level comparison between the healthy participants and the patients was done with the Wilcox rank sum test. Enterotype assignment, encoded as “ET_B” (*Bacteroides* enriched), “ET_P” (*Prevotella* enriched), and “ET_F” (Firmicutes enriched), were generated using the classification tool (http://enterotypes.org/) and are independent of *de novo* clustering. Differences were considered significant if the *p*-value was <0.05 using the chi-square test. Figures were prepared using GraphPad Prism software, version 6.

## Results

### Characteristics of the study population

Eleven subjects were diagnosed with acute schistosomiasis based on positive *Schistosoma* egg and IHA tests; 15 participants were healthy subjects from the same area (controls). All participants were between 39 and 68 years old, and they were divided according to age into the ≥50-year-old group and the <50-year-old group. They could be divided into three groups based on the serum IHA test results (Table 1).

### Microbial communities by host infection status

**Figure 1:**
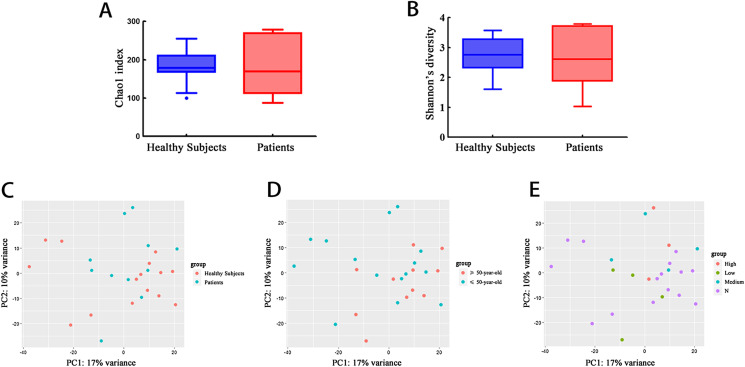
Relative abundance of bacterial phyla in infected patients (*n* = 11) and healthy controls (*n* = 15). (A) No significant differences were observed in the mean community richness as estimated by the Chao1 index. (B) No significant differences were observed in the mean community richness as estimated by the Shannon diversity index. (C) Principal coordinate analysis of the microbial communities in healthy controls and acute schistosomiasis patients using unweighted UniFrac distances by infection status (C), by age group (D), and indirect hemagglutination antibody (IHA) levels (E).

A total of 939,453 quality-filtered reads were obtained for an average of 34,795 reads per sample. Reads were clustered into 419 unique genera and assigned to 27 bacterial phyla. The Chao1 index – which is used to estimate the total number of observed species in each community – indicated that there was no significant difference between the healthy controls and infected subjects (Fig. 1A). The Shannon diversity index – which indicates the diversity of species in every sample – was also not significantly different between the assessed groups (Fig. 1B). Principal coordinate analysis (PCoA) of the fecal microbial communities showed strong clustering of the samples by individual rather than infection status, age group, or IHA test result (Figs. 1C–1E). This suggests that the microbial community composition in each subject remained relatively stable regardless of *S. japonicum* infection status.

### Differentially abundant taxa

**Figure 2:**
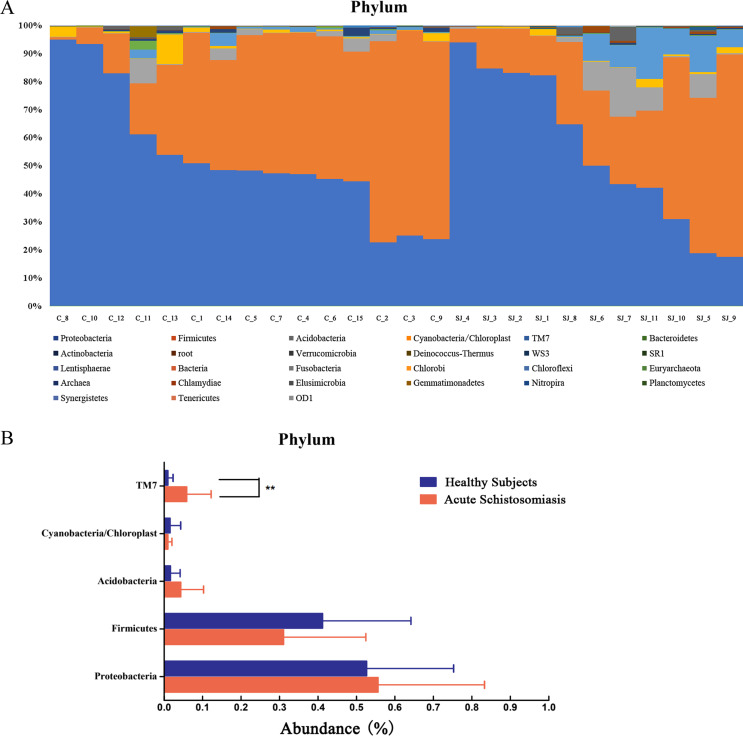
Differences in microbial community structure at the phylum level. (A) Proteobacteria and Firmicutes were the dominant phyla in both groups. (B) Significant difference in the relative abundance of TM7 between healthy controls and infected subjects.

Proteobacteria and Firmicutes were the dominant phyla in the fecal microbial communities of both groups (Fig. 2A). The mean relative abundance of the top five phyla in infected subjects and healthy controls based on the Wilcoxon rank sum statistical comparison were: Proteobacteria (52.7% versus 55.6% in infected subjects and healthy controls, respectively, *p* = 0.38, *p*-value > 0.05), Firmicutes (41.2% vs. 31.1% in infected subjects and healthy controls, respectively, *p* = 0.13, *p*-value > 0.05), Acidobacteria (1.6% vs. 4.3% in infected subjects and healthy controls, respectively, *p* = 0.06, *p*-value > 0.05), Cyanobacteria/Chloroplast (1.5% vs. 1.0% in infected subjects and healthy controls, respectively, *p* = 0.27, *p*-value > 0.05), and TM7 (which is synonymous with Saccharibacteria [7] based on genome comparison [11, 42] and is referred to as TM7 in this study for comparison with the reference) (1.0% vs. 5.9% in infected subjects and healthy controls, respectively, *p* = 0.003, *p*-value < 0.01) (Fig. 2B).

**Figure 3:**
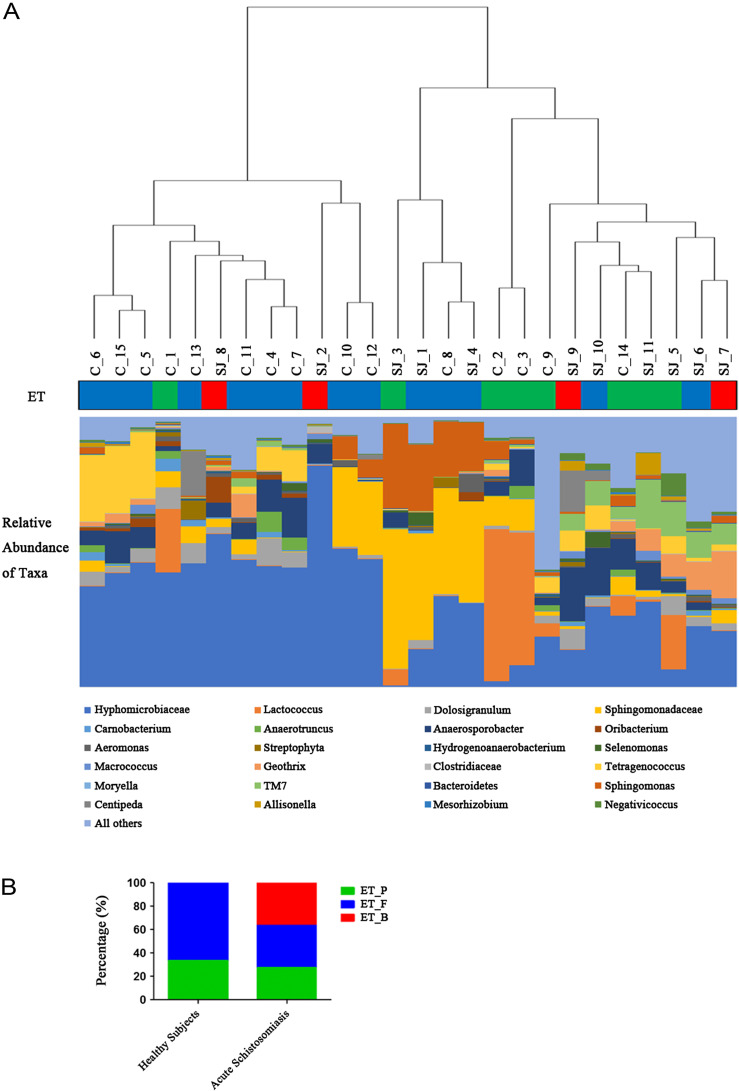
Bar chart showing the composition of the relative abundant bacterial groups and enterotype of each subject at the genus level. (A) Relative abundant bacterial of taxa in each subject according to the Euclidean distance metric and “Complete” linkage of cluster. (B) distribution of healthy subjects and acute schistosomiasis patients over three enterotypes: *Prevotella* (ET_P, *n* = 8), Firmicutes (ET_F, *n* = 14), and *Bacteroides* (ET_B, *n* = 4). Chi-square test for independence: *n* = 26, *χ*^2^ = 6.61, *p* = 0.037.

At the genus level, there were seven differentially abundant genera. Five genera (Comamonas, Psychrobacter, Clostridium, Veillonella, and Butyricimonas) had significantly lower relative abundance in infected patients than in the healthy controls (adjusted *p*-value < 0.05); meanwhile, two genera (Methylophilus and Turicibacter) had significantly higher relative abundance in infected patients than in the healthy controls (adjusted *p*-value < 0.05) (Fig. 3A and Table 2).

**Table 2:** The relative abundance of differentially abundant taxa based on DESeq2 analysis.

Phylum	Class	Order	Family	Genus	Average abundance		Adjusted *p*-value	Sig. higher with Schisto	Sig. lower with Schisto
					Healthy subjects	patients			
Proteobacteria	Betaproteobacteria	Methylophilales	Methylophilaceae	*Methylophilus*	1.93E-05	2.07E-05	1.62E-03		Y
Firmicutes	Erysipelotrichia	Erysipelotrichales	Erysipelotrichaceae	*Turicibacter*	3.21E-05	8.51E-04	2.98E-02		Y
Bacteroidetes	Bacteroidia	Bacteroidales	Porphyromonadaceae	*Butyricimonas*	6.17E-03	9.73E-04	7.96E-03	Y	
Proteobacteria	Betaproteobacteria	Burkholderiales	Comamonadaceae	*Comamonas*	2.08E-03	2.26E-06	1.62E-03	Y	
Proteobacteria	Gammaproteobacteria	Pseudomonadales	Moraxellaceae	*Psychrobacter*	1.07E-03	2.26E-06	1.37E-02	Y	
Firmicutes	Erysipelotrichia	Erysipelotrichales	Erysipelotrichaceae	*Clostridium*	1.63E-03	1.22E-03	1.37E-02	Y	
Firmicutes	Negativicutes	Selenomonadales	Veillonellaceae	*Veillonella*	2.32E-02	9.52E-03	1.25E-02	Y	

### Detection of the gut microbial enterotypes in healthy subjects and patients

Some reports have indicated that enterotypes provide a new perspective for microbial markers related to certain diseases or specific host traits [54]. Based on online enterotype classification and compared with large-scale projects such as MetaHIT and HMP [2, 33], enterotypes were significantly differentially distributed between healthy subjects and acute schistosomiasis patients (chi-square test for independence: *n* = 26, *χ*^2^ = 6.61, *p* = 0.037), with the patients harboring the unique *Bacteroides* enterotype. A comparison between healthy subjects and acute schistosomiasis patients was made in Figure 3B.

## Discussion

This study is the first description of gut microbiota changes in *S. japonicum-*infected patients in an epidemic area in China by high-throughput 16S rDNA gene sequencing. Comparing the alpha diversity index using Kruskal–Wallis tests, the Chao1 index and Shannon diversity index, no significant differences were observed between patients and healthy subjects. Although these data were the same as for subjects infected with *S. haematobium* and controls [1], the relatively small sample size was a limitation of our study, and may have affected the statistical power to identify minor changes in the fecal microbiota following *S. japonicum* infection. However, our most intriguing finding was that there was a higher proportion of TM7 and a *Bacteroides*-rich enterotype in patients with acute *S. japonicum* infection than in healthy controls.

As a unique phylum, TM7, which exhibited an increase in relative abundance in acute *S. japonicum* infected patients, has not previously been linked to schistosome infection [47]. TM7 is globally distributed and is often associated with human inflammatory mucosal diseases. In particular, TM7 is a kind of oral resident bacteria [14] that is dominant in cases of intra-oral halitosis [48]. Cultivation of a human-associated TM7 phylotype revealed a complete lack of capacity for amino acid biosynthesis; this suggests a potential for immune suppression through the repression of TNF-alpha production in macrophages [26]. Other studies have shown that periodontal disease is a risk factor for human colorectal cancer [41], and have identified orally associated bacteria as biomarkers for cancer. Therefore, compared with our data, we suggest that the increase in the relative abundance of TM7 may be a novel biomarker associated with *S. japonicum* infection.

In our study, Proteobacteria and Firmicutes were the most altered phyla in response to *S. japonicum* infection; this is consistent with previous studies [37]. An increasing amount of data identifies Proteobacteria as a possible microbial signature of disease [43]. Proteobacteria are present in various human body sites, including the skin, oral cavity, tongue, vaginal tract, and gut [12], and were found to be correlated with the genesis of endotoxemia and in the development of metabolic disorders [49]. It is possible that alterations in pH, bile flow, ratio of obligate anaerobes to facultative anaerobes, and intestinal hormone levels influence the abundance of Proteobacteria in the feces [27]. Specifically, some researchers consider *Methylophilus* to be an aerobic, methanol-utilizing bacteria [19] that utilizes a number of organic carbon compounds for growth; additionally, it has traditionally been regarded as one of the most important saccharolytic species and expresses multiple anti-oxidative enzymes [18]. In addition, it has also been linked to the stimulation of TNF production *in vitro* from peripheral blood mononuclear cells of healthy patients, as well as a higher circulating IgG response in patients affected by inflammatory bowel disease [5, 18]. This might indicate that the abundance of *Methylophilus* in infected patients in our study is the result of an inflammatory response to acute schistosomiasis. We also found that the relative abundance of *Comamonas* and *Psychrobacter* were significantly decreased in infected patients. *Comamonas* is an obligate aerobe [51]. *Psychrobacter* is a kind of cold adapted bacteria that can help to moderate temperature for other bacteria and enzymes [15, 17] to maintain a suitable environment for infection. The alterations in the relative abundance of these three genera of Proteobacteria suggest that *S. japonicum* infection induces a systemic inflammation response, possibly by potentially highly efficient xenobiotic metabolizing species.

The relative abundance of *Turicibacter* was increased in infected patients, which suggests it may be involved in the development of systemic inflammation through alterations in immune cell activation [24]. However, the relative abundance of *Clostridium*, *Butyricimonas* and *Veillonella* were decreased in infected patients. Some studies have reported that decreased levels of *Clostridium* are correlated with total cholesterol levels [22], which suggests that these taxa may play a role in the infection through effects on lipid metabolism. In addition, the relative abundance of *Butyricimonas* and *Veillonella* have been reported to be negatively correlated with the severity of a number of diseases [20]. Thus, the alterations to the relative abundance of these taxa show that the microbiota is correlated with changes in metabolism and immune response during *S. japonicum* infection.

The identification of gut microbial clusters in the two groups was confirmed in our study. There are at least three gut microbial enterotypes, dominated by *Bacteroides, Faecalibacterium* (classified as *Ruminococcus* at the time), or *Prevotella* [13]. Similarly, we found one cluster of fecal communities that was distinguished by *Bacteroides* levels in acute schistosomiasis patients. This enterotype has been linked to alterations in nutrient processing, and also corresponds with the presence of genes that code for enzymes such as proteases, hexoaminidases, and galactosidases [29]. Growing evidence supports the notion that *Bacteroides* activate an infectious response in the intestine [6] and they were significantly more abundant in a mouse model of *S. mansoni* infection [30]. Based on the altered immune response in acute *S. japonicum* infection [56] and the data from mouse gut microbial modulation after infection with *S. japonicum* cercaria [57], future studies should examine whether the shift in enterotypes is associated with the *Bacteroides* enterotype as a possible biomarker for acute schistosomiasis [21].

## Conclusions

In this study, we identified differentially abundant taxa in the gut microbiota of infected subjects and healthy controls, which may be associated with inflammation in patients with acute schistosomiasis. Further studies are required to unveil the functional roles conserved in these significantly altered taxa, to determine the interspecies interactions with the gut microbiota of patients with acute schistosomiasis, to determine the interactions between gut microbiota members and *S. japonicum,* as well as with host, and to determine the link between systemic inflammation, alterations in fecal microbiota structure, and disease activity.

## Data availability statement

The raw sequencing data from 16S rRNA gene sequencing used in this study are available in the short reads archive (SRA) database (accession number PRJNA625383).

## Author contributions

Yanyan Jiang and Jianping Cao designed the study. Jianping Cao contributed reagents and materials. Yanyan Jiang, Zhongying Yuan, Yujuan Shen, and Shengkui Cao performed the experiments. Yanyan Jiang, Makedonka Mitreva, Bruce A. Rosa, John Martin, and Jianping Cao analyzed and interpreted the data. Yanyan Jiang wrote the manuscript. Jianping Cao, Makedonka Mitreva, Bruce A. Rosa, and Yanjiao Zhou revised the manuscript. The authors declare that there are no conflicts of interest.

## Funding

This study was funded by the National Natural Science Foundation of China (Nos. 81772225 and 81971969 to JC), the National Key Research and Development Program of China (No. 2017YFD0501300 to YJ) and the Chinese Special Program for Scientific Research of Public Health (No. 201502021 to JC). The funders had no role in study design, data collection and analysis, decision to publish, or preparation of the manuscript.

## Supplementary material

Supplementary material is available at https://www.parasite-journal.org/10.1051/parasite/2020074/olm *Table S1*. Bacterial taxa associated with healthy subjects and patients at the phylum and genus levels.
