# Supplementary material for: Alteration of the fecal microbiota in Chinese patients with Schistosoma japonicum infection
Source: Parasite. 2021 Jan 8;28:1. doi: 10.1051/parasite/2020074 (PMC7792497; doi:10.1051/parasite/2020074)
Supplement: Supplementary file 1 — Table S1. Bacterial taxa associated with healthy subjects and patients at the phylum and genus levels. [file parasite-28-1-s1.pdf]

Supplement Table S1

Sample name	Tag Numbers	Unique Genera
C_1	34998	170
C_2	36284	218
C_3	36711	161
C_4	34499	155
C_5	34997	164
C_6	32341	198
C_7	32061	173
C_8	39589	94
C_9	35189	168
C_10	37712	84
C_11	33040	231
C_12	37270	153
C_13	33611	138
C_14	34119	243
C_15	33190	170
SJ_1	38177	121
SJ_2	34598	92
SJ_3	39670	135
SJ_4	37744	80
SJ_5	33438	258

SJ_6	32311	254
SJ_7	34587	269
SJ_8	36130	173
SJ_9	30387	111
SJ_10	32286	75
SJ_11	33437	63
